# Impact of severe immunoparesis degree and duration on progression-free survival in newly diagnosed multiple myeloma: a retrospective cohort study of 258 patients

**DOI:** 10.3389/fonc.2026.1831800

**Published:** 2026-08-06

**Authors:** Linlin Tian, Yuan Zhang, Jiong Chen, Guoqing Lv, Xiankai Liu, Yuanyuan Cui, Yan Guo, Sun Wu

**Affiliations:** 1 Department of Hematology, the First Affiliated Hospital of Henan Medical University, Weihui, Henan, China; 2 Key Laboratory for Lymphoma Molecular Diagnosis and Treatment in Xinxiang City, Weihui, Henan, China; 3 Key Laboratory for Leukemia Molecular Diagnosis and Treatment in Xinxiang City, Weihui, Henan, China; 4 Department of Pathology, The First Affiliated Hospital of Henan Medical University, Weihui, Henan, China

**Keywords:** immunoparesis, multiple myeloma, prognosis, progression-free survival, tumor load

## Abstract

**Objective:**

To investigate the relationship between the degree and duration of immunoparesis at diagnosis and poor prognosis in patients with newly diagnosed multiple myeloma (NDMM).

**Methods:**

A retrospective analysis was conducted on the clinical data of 258 patients with NDMM, including the status and duration of immunoparesis and the influence of other factors on prognosis.

**Results:**

91.5% of NDMM had varying degrees of immunoparesis. The median progression-free survival (PFS) was significantly shorter in the severe immunoparesis group than in the non-severe immunoparesis group (25 months vs. 34 months, P = 0.0149). The median PFS was significantly shorter in the groups with severe immunoparesis lasting ≥ 3, ≥ 6, and ≥ 12 months than in the group with non-severe immunoparesis lasting (P ≤0.05). Osteolytic lesions, 17p deletion/mutation, hemoglobin < 85g/L, and severe immunoparesis lasting ≥ 12 months were independent poor prognostic factors for PFS (all P < 0.05). Circulating plasma cells ≥ 2% and osteolytic lesions were independent poor prognostic factors for overall survival (OS) (all P < 0.05).

**Conclusion:**

Immunoparesis at diagnosis and its duration are poor prognostic factors for PFS in patients with NDMM.

## 1 Introduction

Multiple myeloma (MM) is the third most common hematological malignancy and remains incurable (1). A hallmark of the disease is plasma cell dysfunction, leading to immunoparesis, hypogammaglobulinemia, and increased susceptibility to infection (2, 3). Immunoparesis is defined as a condition in which one or more uninvolved polyclonal immunoglobulin (Ig) levels fall below the lower limit of the normal range. It is observed at all stages of multiple myeloma, including monoclonal gammopathy of undetermined significance and smoldering multiple myeloma, and has been associated with disease progression (4–7). Immunoparesis is observed in more than half of patients with newly diagnosed multiple myeloma (8–12). In recent years, the prognostic role of immunoparesis has garnered increasing attention. However, the association between baseline immunoparesis and outcomes in NDMM remains controversial. Moreover, previous studies have primarily focused on the presence of immunoparesis at diagnosis without systematically evaluating its degree or duration. In this study, we analyzed the correlation between the degree and duration of immunoparesis at diagnosis and survival outcomes in NDMM, aiming to provide further insight into the prognostic implications of immunoparesis in this population.

## 2 Materials and methods

### 2.1 Patients and treatment

#### 2.1.1 Clinical data

This retrospective study included 258 patients diagnosed with NDMM at the First Affiliated Hospital of Henan Medical University between April 2018 and September 2025. All patients met the diagnostic criteria of the International Myeloma Working Group (IMWG) (13) and were over 18 years of age. All patients received bortezomib-based induction therapy. The regimens were categorized as follows: VRd (bortezomib + lenalidomide + dexamethasone), Vd (bortezomib + dexamethasone), VCd (bortezomib + cyclophosphamide + dexamethasone), VTd (bortezomib + thalidomide + dexamethasone), VMP (bortezomib + melphalan + prednisone), DVd (daratumumab + bortezomib + dexamethasone), DVCd (daratumumab + bortezomib + cyclophosphamide + dexamethasone), DRVd (daratumumab + lenalidomide + bortezomib + dexamethasone), and DPVd (daratumumab + pomalidomide + bortezomib + dexamethasone). Baseline data were retrospectively collected from electronic medical records. The data included gender, age, Durie-Salmon (DS) stage, International Staging System (ISS) stage, Revised International Staging System (R-ISS) stage, M-protein type, cytogenetics, bone marrow examination, serum protein electrophoresis, urine protein electrophoresis, lactate dehydrogenase (LDH), hemoglobin (Hb), calcium (Ca), albumin (Alb), serum creatinine (Scr), imaging findings, circulating plasma cell ratio, treatment regimen, and treatment response.

#### 2.1.2 Assessment of immunoparesis

Serum immunoglobulin G (IgG), immunoglobulin A (IgA), and immunoglobulin M (IgM) levels were measured by immunoturbidimetry at diagnosis, at the beginning of each induction treatment cycle, and every two months during consolidation or maintenance therapy. Monitoring continued until disease progression, death, or discontinuation of follow-up. In cases of disease progression, the same immunoglobulin assessment protocol used at diagnosis was repeated. The lower limits of the normal ranges for IgG, IgA, and IgM were 7.0 g/L, 0.7 g/L, and 0.4 g/L, respectively.

No immunoparesis was defined as a state in which all uninvolved immunoglobulins remained within the normal reference range. Mild immunoparesis was defined as a condition in which at least one uninvolved immunoglobulin level fell below the lower limit of normal but remained ≥ 50% of the lower limit of the normal reference range. Severe immunoparesis (12, 14) was defined as a condition in which at least one uninvolved immunoglobulin level was < 50% of the lower limit of the normal reference range. Based on severity, patients were categorized into the non-severe immunoparesis group (including mild immunoparesis and no immunoparesis) and the severe immunoparesis group. According to the duration of severe immunoparesis, patients were further subdivided into groups with non-severe immunoparesis lasting group and those with severe immunoparesis lasting ≥ 3 months, severe immunoparesis lasting ≥ 6 months, and severe immunoparesis lasting ≥ 12 months.

#### 2.1.3 Assessment of osteolytic lesions

At initial diagnosis, all patients underwent either emission computed tomography (ECT) or conventional radiographic examination (X-ray). Osteolytic lesions were defined according to the International Myeloma Working Group (IMWG) criteria (15) as imaging-visible bone defects with cortical destruction and a punched-out or moth-eaten appearance without a sclerotic margin.

### 2.2 Response and outcome

Treatment response and disease progression were evaluated according to the IMWG criteria (15). The primary endpoint was PFS, defined as the time from treatment initiation to disease progression or death from any cause, whichever occurred first. The secondary endpoint was OS, defined as the time from treatment initiation to death from any cause. Follow-up concluded on September 30, 2025.

### 2.3 Statistical analysis

Categorical variables were expressed as frequencies or percentages and compared by χ^2^ test. Survival curves were estimated using the Kaplan-Meier method, and differences between groups were compared using the log-rank test. Univariate and multivariate analyses were performed using Cox proportional hazards regression to identify factors associated with PFS and OS. P < 0.05 was considered statistically significant. Statistical analyses were performed using IBM SPSS Statistics 26.0 and GraphPad Prism 10.1.2.

## 3 Results

### 3.1 Patient characteristics

A total of 258 patients with NDMM were enrolled from April 2018 to September 2025. Of these, 22 patients (8.5%) had no immunoparesis, 75 (29.1%) had mild immunoparesis, and 161 (62.4%) had severe immunoparesis. The male-to-female ratio was 1.21 (141:117). The median age was 65 years (range: 37-87). All patients received induction therapy with novel agents; 27 (10.5%) patients received therapy that included daratumumab. Following induction therapy, 21 patients (8.1%) underwent autologous stem cell transplantation (ASCT). Baseline characteristics are summarized in **Table 1**. Significant differences between the non-severe and severe immunoparesis groups were observed in DS stage, ISS stage, R-ISS stage, M-protein type, hemoglobin <85 g/L, albumin <35 g/L, 1q21 gain/amplification, and amyloidosis (all P < 0.05; **Table 1**).

**Table 1:** Baseline characteristics.

Characteristics	Non-severe immunoparesis(n=97)	Severe immunoparesis (n=161)	χ^2^	*P*
Sex, n(%)			1.073	0.300
Male	49(50.5%)	92(57.1%)		
Female	48(49.5%)	69(42.9%)		
Age > 65 years, n(%)			0.065	0.799
Yes	43(44.3%)	74(46.0%)		
No	54(55.7%)	87(54.0%)		
DS, n(%)			15.988	<0.001
I stage	11(11.3%)	5(3.1%)		
II stage	19(19.6%)	13(8.1%)		
III stage	67(69.1%)	143(88.8%)		
ISS, n(%)			7.788	0.020
I stage	29(29.9%)	25(15.5%)		
II stage	28(28.9%)	61(37.9%)		
III stage	40(41.2%)	75(46.6%)		
R-ISS, n(%)			7.063	0.029
I stage	22(22.7%)	17(10.6%)		
II stage	61(62.9%)	120(74.5%)		
III stage	14(14.4%)	24(14.9%)		
M protein, n(%)			12.652	0.005
IgG	37(38.1%)	91(56.5%)		
IgA	21(21.6%)	33(20.5%)		
Light chain	33(34.0%)	26(16.1%)		
Others	6(6.2%)	11(6.8%)		
Induction therapy, n(%)			3.112	0.539
VRd	30(30.9%)	58(36.0%)		
VCd	28(28.9%)	54(33.5%)		
Vd	18(18.6%)	21(13.0%)		
VTd	5(5.2%)	4(2.5%)		
Others	16(16.5%)	24(14.9%)		
Induction therapy with Daratumumab, n(%)			0.603	0.438
Yes	12(12.4%)	15(9.3%)		
No	85(87.6%)	146(90.7%)		
Osteolytic lesions, n(%)			2.078	0.149
Yes	75(77.3%)	136(84.5%)		
No	22(22.7%)	25(15.5%)		
LDH > 250U/L, n(%)			0.003	0.954
Yes	16(16.5%)	27(16.8%)		
No	81(83.5%)	134(83.2%)		
Hb < 85g/L, n(%)			10.975	0.001
Yes	33(34.0%)	89(55.3%)		
No	64(66.0%)	72(44.7%)		
Scr ≥ 177umol/L, n(%)			1.373	0.241
Yes	28(28.9%)	36(22.4%)		
No	69(71.1%)	125(77.6%)		
Ca > 2.65mmol/L, n(%)			2.437	0.119
Yes	9(9.3%)	26(16.1%)		
No	88(90.7%)	135(83.9%)		
Alb < 35g/L, n(%)			7.286	0.007
Yes	38(39.2%)	91(56.5%)		
No	59(60.8%)	70(43.5%)		
ASCT, n(%)			0.002	0.961
Yes	8(8.2%)	13(8.1%)		
No	89(91.8%)	148(91.9%)		
1q21gain/amplification, n(%)			5.837	0.016
Yes	35(36.1%)	83(51.6%)		
No	62(63.9%)	78(48.4%)		
17p deletion/mutation, n(%)			0.575	0.448
Yes	8(8.2%)	18(11.2%)		
No	89(91.8%)	143(88.8%)		
IGH rearrangement, n(%)			1.715	0.190
Yes	33(34.0%)	68(42.2%)		
No	64(66.0%)	93(57.8%)		
Extramedullary disease, n(%)			0.836	0.361
Yes	16(16.5%)	20(12.4%)		
No	81(83.5%)	141(87.6%)		
Amyloidosis, n(%)			13.316	<0.001
Yes	14(14.4%)	4(2.5%)		
No	83(85.6%)	157(97.5%)		
Circulating plasma cells ≥ 2%, n(%)			1.790	0.181
Yes	6(6.2%)	18(11.2%)		
No	91(93.8%)	143(88.8%)		

### 3.2 Survival outcome

**Figure 1:**
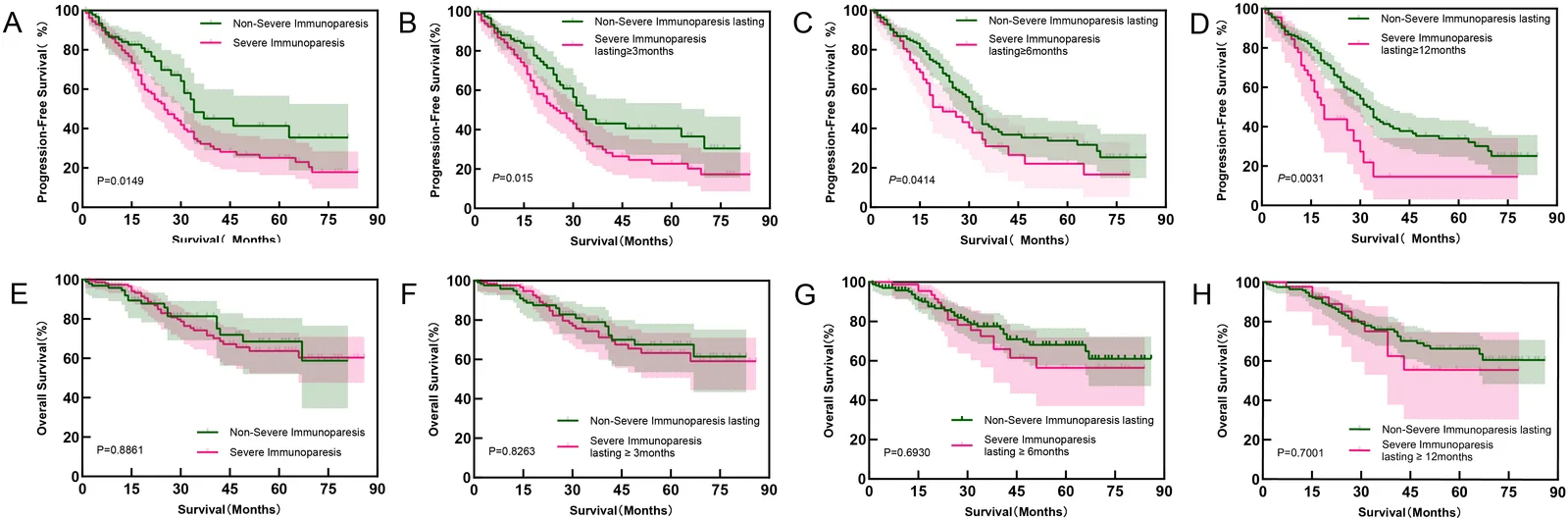
PFS **(A)**/OS **(E)** survival curves of the severe immunoparesis group and the non-severe immunoparesis group; PFS **(B)**/OS **(F)** survival curves for the group with severe immunoparesis lasting ≥ 3 months and the group with non-severe immunoparesis lasting; PFS **(C)**/OS **(G)** curves for the group with severe immunoparesis lasting ≥ 6 months and the group with non-severe immunoparesis lasting; PFS **(D)**/OS **(H)** curves for the group with severe immunoparesis lasting ≥ 12 months and the group with non-severe immunoparesis lasting.

The median follow-up time was 23 months (range, 1–86 months). By the end of follow-up, the mortality rate was 20.9% (54/258). The median PFS was 25 months in the severe immunoparesis group and 34 months in the non-severe immunoparesis group (P = 0.0149; **Figure 1A**). For patients with severe immunoparesis lasting ≥ 3 months, the median was 25 months, compared with 33 months in the group with non-severe immunoparesis lasting (P = 0.015; **Figure 1B**). For those with severe immunoparesis lasting ≥ 6 months, the median PFS was 22 months versus 31 months (P = 0.0414; **Figure 1C**). For those with severe immunoparesis lasting ≥ 12 months, the median PFS was 19 months versus 33 months (P = 0.0031; **Figure 1D**).

Median OS was not reached in either the severe or non-severe immunoparesis groups (P = 0.8861; **Figure 1E**). Similarly, no significant differences in OS were observed between the groups with severe immunoparesis lasting ≥ 3, ≥ 6, or ≥ 12 months and the corresponding non-severe immunoparesis lasting groups (all P > 0.05; **Figures 1F–H**).

### 3.3 Univariate and multivariate analysis

Univariate Cox regression analysis indicated that circulating plasma cells ≥ 2%, osteolytic lesions, hemoglobin < 85 g/L, albumin < 35 g/L, 17p deletion/mutation, severe immunoparesis, and severe immunoparesis lasting ≥ 3, ≥ 6, or ≥ 12 months were poor prognostic factors for PFS (all P < 0.05; **Table 2**). For OS, circulating plasma cells ≥ 2%, osteolytic lesions, hemoglobin < 85 g/L, albumin < 35 g/L, and IGH rearrangement were poor prognostic factors (all P < 0.05; **Table 3**).

**Table 2:** Univariate analysis for progression-free survival.

Factor	Univariate analysis		
	*P*	HR	95%CI
ASCT	0.218	0.533	0.196-1.450
Extramedullary disease	0.230	1.362	0.823-2.255
Amyloidosis	0.619	0.796	0.324-1.956
Circulating plasma cells ≥ 2%	0.006	2.155	1.245-3.729
Age > 65 years	0.390	1.175	0.814-1.696
Male	0.264	0.809	0.558-1.173
Osteolytic lesions	0.005	2.073	1.250-3.437
LDH > 250U/L	0.102	1.449	0.928-2.260
Hb < 85g/L	0.017	1.562	1.084-2.250
Scr ≥ 177umol/L	0.356	1.222	0.798-1.872
Ca > 2.65mmol/L	0.065	1.592	0.972-2.607
Alb < 35g/L	0.434	1.156	0.804-1.662
1q21gain/amplification	0.832	1.040	0.722-1.498
17p deletion/mutation	<0.001	2.615	1.591-4.297
IGH rearrangement	0.095	1.363	0.947-1.961
Severe immunoparesis	0.017	1.648	1.093-2.486
Severe immunoparesis lasting ≥ 3 months	0.017	1.578	1.085-2.294
Severe immunoparesis lasting ≥ 6months	0.045	1.473	1.009-2.150
Severe immunoparesis lasting ≥ 12months	0.004	1.904	1.229-2.949

**Table 3:** Univariate and multivariate analysis for overall survival.

Factor	Univariate analysis			Multivariate analysis		
	*P*	HR	95%CI	*P*	HR	95%CI
ASCT	0.251	0.045	0.000-8.957	–	–	–
Extramedullary disease	0.959	1.021	0.461-2.260	–	–	–
Amyloidosis	0.817	1.148	0.357-3.639	–	–	–
Circulating plasma cells ≥ 2%	0.001	3.155	1.575-6.320	0.049	2.054	1.002-4.209
Age > 65 years	0.561	0.847	0.483-1.484	–	–	–
Male	0.819	1.065	0.621-1.827	–	–	–
Osteolytic lesions	0.027	2.611	1.116-6.110	0.030	2.596	1.094-6.161
LDH > 250U/L	0.527	1.238	0.638-2.402	–	–	–
Hb < 85g/L	0.005	2.218	1.276-3.857	0.075	1.724	0.946-3.142
Scr ≥ 177umol/L	0.563	1.197	0.651-2.202	–	–	–
Ca > 2.65mmol/L	0.719	1.148	0.541-2.436	–	–	–
Alb < 35g/L	0.032	1.819	1.052-3.145	0.239	1.435	0.787-2.617
1q21gain/amplification	0.422	0.803	0.470-1.372	–	–	–
17p deletion/mutation	0.468	1.343	0.606-2.977	–	–	–
IGH rearrangement	0.035	1.784	1.043-3.054	0.103	1.573	0.912-2.712
Severe immunoparesis	0.886	1.042	0.591-1.836	–	–	–
Severe immunoparesis lasting ≥ 3months	0.827	1.062	0.620-1.817	–	–	–
Severe immunoparesis lasting ≥ 6months	0.694	1.123	0.630-2.000	–	–	–
Severe immunoparesis lasting ≥ 12months	0.701	1.145	0.574-2.281	–	–	–

Multivariate Cox regression analysis (**Table 4**) demonstrated that osteolytic lesions, 17p deletion/mutation, hemoglobin < 85 g/L, and severe immunoparesis lasting ≥ 12 months were independently associated with shorter PFS (all P < 0.05). For OS (**Table 3**), circulating plasma cells ≥ 2% and osteolytic lesions were identified as independent poor prognostic factors (all P < 0.05).

**Table 4:** Multivariate analysis for progression-free survival.

Factor	Multivariate analysis(Model-1)			Multivariate analysis(Model-2)			Multivariate analysis(Model-3)			Multivariate analysis(Model-4)		
	*P*	HR	95%CI	*P*	HR	95%CI	*P*	HR	95%CI	*P*	HR	95%CI
Circulating plasma cells ≥ 2%	0.129	1.547	0.881-2.716	0.141	1.530	0.869-2.695	0.192	1.471	0.823-2.629	0.175	1.485	0.838-2.631
Osteolytic lesions	0.016	1.879	1.125-3.140	0.017	1.872	1.119-3.133	0.015	1.892	1.132-3.164	0.017	1.870	1.118-3.125
17p deletion/mutation	<0.001	2.688	1.613-4.481	<0.001	2.764	1.654-4.620	<0.001	2.671	1.604-4.450	<0.001	2.538	1.515-4.251
Hb < 85g/L	0.036	1.514	1.027-2.232	0.026	1.545	1.054-2.266	0.012	1.620	1.110-2.365	0.010	1.643	1.128-2.394
Severe immunoparesis	0.114	1.414	0.921-2.173	–	–	–	–	–	–	–	–	–
Severe immunoparesis lasting ≥ 3 months	–	–	–	0.062	1.444	0.982-2.124	–	–	–	–	–	–
Severe immunoparesis lasting ≥ 6months	–	–	–	–	–	–	0.209	1.289	0.867-1.914	–	–	–
Severe immunoparesis lasting≥ 12months	–	–	–	–	–	–	–	–	–	0.020	1.702	1.086-2.666

Circulating plasma cells ≥ 2%, osteolytic lesions, 17p deletion/mutation and Hb < 85g/L were included as covariates in models 1-4. Severe immunoparesis, severe immunoparesis lasting ≥ 3 months, severe immunoparesis lasting ≥ 6 months, severe immunoparesis lasting ≥ 12 months were included respectively as covariates in models 1-4.

## 4 Discussion

Most MM patients exhibit varying degrees of immunoparesis at initial diagnosis (8–10, 16). In our study, 91.4% of patients presented with immunoparesis of varying severity, and 62.4% had severe immunoparesis. Patients with severe immunoparesis were more likely to present with anemia and hypoproteinemia, which is partially consistent with previous studies (9, 10, 17, 18).

The relationship between M-protein isotype and immunoparesis severity remains unclear. Heaney et al. (18) reported variations in immunoparesis severity among different M-protein subtypes, with IgD, IgA, and lambda light-chain MM showing the most pronounced suppression, although the overall incidence of immunoparesis did not differ significantly by isotype. Similarly, Pruzanski et al. (19) found no significant difference in incidence across subtypes. In contrast, Kyle et al. (20) reported a higher frequency of immunoparesis in IgA MM compared with IgG MM (P = 0.004). In our study, IgG MM was significantly associated with severe immunoparesis (P = 0.005), consistent with a single-center study from China (21). These findings suggest that M-protein isotype may influence the degree of immunoparesis, although the underlying mechanisms require further investigation. Whether these associations differ between Eastern and Western populations warrants validation in larger, multi-ethnic cohorts.

Our finding that severe immunoparesis was associated with shorter PFS is in line with several earlier studies. Sørrig et al. (9) reported that immunoparesis at initial diagnosis was an independent risk factor for PFS but not OS in a Danish cohort. Chen et al. (22) observed similar results in patients treated with novel agents. In the transplant-ineligible population, Dávila et al. (23) found that although 81.2% of patients had immunoparesis at diagnosis, it did not independently predict PFS or OS. In transplant-eligible patients, Lakhwani et al. (16) a high prevalence of immunoparesis (94.5%) but no independent prognostic significance. Conversely, Geng et al. (12) demonstrated that baseline immunoparesis was an independent risk factor for both PFS and OS. Kastritis et al. (10) also identified immunoparesis as an independent predictor of OS in a large cohort of 1,755 patients.

Immunoparesis is dynamic, and its evolution during treatment may reflect disease response and immune reconstitution. Yan et al. (21) found that improvement in immunoparesis correlated with depth of response and PFS benefit. Chen et al. (24) reported that persistent severe immunoparesis predicted inferior survival in non-transplant patients. Yin et al. (25) observed that 38.4% of patients still had severe immunoparesis after achieving optimal response, and those with persistent severe immunoparesis had shorter PFS. Zhou et al. (26) further showed that patients with sustained improvement of immunoparesis for more than 12 months had significantly better outcomes. Lakhwani et al. (16) further demonstrated that recovery from immunoparesis was independently associated with favorable PFS in transplant-eligible NDMM patients receiving continuous intensive therapy, underscoring the prognostic value of longitudinal immune monitoring during treatment. Similarly, Chakraborty et al. (27) reported that 91% of 258 patients with relapsed MM presented with immunoparesis at the time of recurrence, among whom 39.9% exhibited severe immunoparesis, and this was significantly correlated with shorter PFS. In our study, 17.4% of patients had severe immunoparesis persisting beyond 12 months, which was associated with shorter PFS. These data underscore the importance of longitudinal immunoparesis assessment in risk stratification.

The relationship between cytogenetic abnormalities and immunoparesis remains an area of active investigation. Caro et al. (14), reported that immunoparesis was more frequent in patients with high-risk cytogenetic features, including t(4;14), t(14;16), and t(14;20), and was prognostically significant in the hyperdiploid subgroup. In contrast, Chakraborty et al. (27) found no association between immunoparesis severity at relapse and baseline cytogenetics. In our cohort, 1q21 gain/amplification was significantly associated with severe immunoparesis, suggesting that specific cytogenetic alterations may contribute to immune suppression. Previous studies have shown that 1q21 amplification is frequently accompanied by overexpression of genes such as *MCL-1*, *IL-6R*, and *CKS1B*, which may promote an immunosuppressive tumor microenvironment (28, 29). Whether these alterations directly impair polyclonal plasma cell function requires further exploration.

From a clinical perspective, the duration of severe immunoparesis may serve as a practical, low-cost biomarker for identifying patients who require closer surveillance or treatment intensification. Those with persistent immunoparesis beyond 12 months, particularly in the presence of high-risk cytogenetics, may benefit from prolonged maintenance therapy or earlier introduction of novel agents. However, given the retrospective and single-center nature of this study, these findings should be interpreted with caution. Larger prospective studies with extended follow-up are needed to confirm our conclusions.

## 5 Conclusions

In summary, the severity and duration of immunoparesis at diagnosis are associated with inferior PFS in patients with NDMM. Dynamic monitoring of immunoparesis may provide valuable prognostic information and guide individualized treatment strategies.

## Data Availability

The raw data supporting the conclusions of this article will be made available by the authors, without undue reservation.
